# Validación Cuestionario FSI-10 y grado de satisfacción con dispositivos de inhaloterapia**

**DOI:** 10.15649/cuidarte.1219

**Published:** 2022-08-06

**Authors:** María-del-Pilar Rojas Laverde, Marcela Roa-Cubaque, Alba Yanira Polanía Robayo, Sandra Patricia Corredor Gamba, Jessica Molina-Franky, Flor Ángela Umbacía Salas

**Affiliations:** 1 Universidad de Boyacá, Tunja, Colombia. Email: pilyrojas@uniboyaca.edu.co Universidad de Boyacá Universidad de Boyacá Tunja Colombia pilyrojas@uniboyaca.edu.co; 2 Universidad de Boyacá, Tunja, Colombia. Email: maroa@uniboyaca.edu.co Universidad de Boyacá Universidad de Boyacá Tunja Colombia maroa@uniboyaca.edu.co; 3 Universidad de Boyacá, Tunja, Colombia. Email: albpolania@uniboyaca.edu.co Universidad de Boyacá Universidad de Boyacá Tunja Colombia albpolania@uniboyaca.edu.co; 4 Universidad de Boyacá, Tunja, Colombia. Email: sancorredor@uniboyaca.edu.co Universidad de Boyacá Universidad de Boyacá Tunja Colombia sancorredor@uniboyaca.edu.co; 5 Universidad de Boyacá, Tunja, Colombia. Email: jsmolina@uniboyaca.edu.co Universidad de Boyacá Universidad de Boyacá Tunja Colombia jsmolina@uniboyaca.edu.co; 6 Universidad de Boyacá, Tunja, Colombia. Email: floumbacia@uniboyaca.edu.co Universidad de Boyacá Universidad de Boyacá Tunja Colombia floumbacia@uniboyaca.edu.co

**Keywords:** Validez de las Pruebas, Reproducibilidad de Resultados, Enfermedad pulmonar, Validity of Tests, Reproducibility of Results, Lung Diseases, Validade do Teste, Reprodutibilidade dos Resultados, Doença Pulmonar

## Abstract

**Introducción:**

El tratamiento farmacológico de la EPOC se realiza progresiva y escalonadamente de acuerdo a la gravedad y puede ser modificado en función de la respuesta al mismo, por lo cual se han desarrollado instrumentos con el fin de evaluar la satisfacción del paciente con los distintos sistemas de inhalación; sin embargo, estos instrumentos han sido diseñados en su mayoría para pacientes con Asma.

**Objetivo:**

Validar el instrumento FSI-10 y determinar el nivel de satisfacción de los dispositivos de inhaloterapia en pacientes con EPOC.

**Materiales y métodos:**

Estudio transversal prospectivo y de validación de contenido, muestreo probabilístico estratificado con afijación proporcional; población de 337 pacientes con diagnóstico EPOC de la provincia Centro del departamento de Boyacá, Colombia. Se evaluó el cuestionario FSI-10 mediante la prueba de esfericidad de Bartlett, Kaiser-Meyer-Olkinyalfade Cronbach.

**Resultados:**

Laprueba de esfericidad resultó estadísticamente significativa, sugiriendo la existencia de correlaciones dentro de la matriz de diez preguntas. La variación total explicable de las diez preguntas que corresponden a la versión del FSI 10 dio cuenta del 81 % de variabilidad. Los pacientes evaluados reportaron un nivel de satisfacción entre bastante a algo con los dispositivos de inhaloterapia.

**Conclusiones:**

Las propiedades psicométricas permiten su utilización en la satisfacción del paciente con dispositivos de medicación inhalada, sin presentar diferencias en la comprensión y teniendo resultado fiables. La satisfacción con los dispositivos de inhaloterapia no es muy alta en la mayoría de la población evaluada.

## Abstract (en)

**Introduction:**

COPD pharmacological treatment is progressively staggered according to severity, which can be modified based on its response. Therefore, instruments have been developed to assess patient satisfaction with different inhaler devices. However, these instruments have been designed mostly for patients with asthma.

**Objective:**

To validate the FSI-10 instrument and assess the degree of satisfaction with inhaler devices in COPD patients.

**Materials and Methods:**

A prospective cross-sectional study for content validation and stratified probability sampling with proportional allocation was conducted with 337 COPD patients in the Central province of the department of Boyacá, Colombia. The FSI-10 questionnaire was assessed using Bartlett’s test of sphericity, Kaiser-Meyer-Olkin test and Cronbach’s alpha.

**Results:**

The test of sphericity was statistically significant, suggesting the presence of a correlation within the ten-question test. Total explained variation of the FSI-10 questions accounted for 81% of the variation. Assessed patients reported a degree of satisfaction between fair to good with the use of inhaler devices.

**Conclusions:**

FSI-10 psychometric properties enable its use in assessing patient satisfaction with inhaler devices with no difference in understanding and reliability results. Satisfaction with inhaler devices is not very high among the majority of the population assessed.

## Resumen

**Introdução:**

O tratamento farmacológico da DPOC é progressivo e escalonado de acordo com a gravidade e pode ser modificado de acordo com a resposta ao tratamento, por isso, foram desenvolvidos instrumentos para avaliar a satisfação do paciente com diferentes sistemas de inalação; no entanto, esses instrumentos foram elaborados principalmente para pacientes com asma.

**Objetivo:**

Validar o instrumento FSI-10 e determinar o nível de satisfação com os dispositivos de inaloterapia em pacientes com DPOC.

**Materiais e métodos:**

Estudo transversal prospectivo e de validação de conteúdo, amostragem probabilística estratificada com alocação proporcional; população de 337 pacientes diagnosticados com DPOC na província central do departamento de Boyacá, Colômbia. O questionário FSI-10 foi avaliado usando o teste de esfericidade de Bartlett, Kaiser-Meyer-Olkin e o alfa de Cronbach.

**Resultados:**

O teste de esfericidade foi estatisticamente significativo, sugerindo a existência de correlações dentro da matriz de dez perguntas. A variação total explicável das dez perguntas correspondentes à versão FSI 10 foi responsável por 81 % da variabilidade. Os pacientes avaliados relataram um nível de satisfação entre bastante e moderada com os dispositivos de inaloterapia.

**Conclusões:**

As propriedades psicométricas permitem seu uso na satisfação do paciente com dispositivos de medicamentos inalados, sem diferenças na compreensão e resultados confiáveis. A satisfação com os dispositivos de inaloterapia não é muito alta na maioria da população avaliada.

## Introducción

La enfermedad pulmonar obstructiva crónica (EPOC) es el problema de salud pública con mayor prevalencia e impacto socioeconómico en el mundo a pesar de ser una enfermedad potencialmente prevenible. Por su elevada frecuencia, su curso clínico progresivo y sus requerimientos asistenciales constituyen un problema médico de primer orden, siendo una de las principales causas de mortalidad a nivel mundial y consumiendo elevados recursos sanitarios^1^. La EPOC es una de las causas de mayor morbilidad y mortalidad en el mundo ocupando el cuarto lugar con un 5.5% de las muertes; en el 2030 constituirá la tercera causa principal de muerte con el 8.6%; tiene además un impacto socio-económico importante, que constituye un problema de salud pública de primer orden a nivel mundial^2^.

Los estudios epidemiológicos realizados en distintas regiones permiten estimar la prevalencia global de EPOC en 10% para individuos mayores de 40 años^3^^,^^4^^,^^5^. Los datos de prevalencia en Latinoamérica provienen de dos estudios: estudio PLATINO (Proyecto Latinoamericano de Investigación en Obstrucción Pulmonar), es un estudio epidemiológico sobre prevalencia de EPOC en individuos de 40 años y más, realizado en cinco ciudades de Latinoamérica: Ciudad de México (México), Sao Pablo (Brasil), Montevideo (Uruguay), Santiago de Chile (Chile), y Caracas (Venezuela); el estudio identificó como afectados de EPOC (relación volumen espiratorio forzado en el primer segundo/capacidad vital forzada < 0,7 posbroncodilatador) a 758, de los que 86 tenían diagnóstico médico previo^6^. Estudio epidemiológico de prevalencia PREPOCOL, desarrollado en cinco ciudades de Colombia, en donde incluyeron un total de 5.539 sujetos, estableciendo una prevalencia de 8,9%^7^. Los datos para el departamento de Boyacá - Colombia se sustentan en el informe de indicadores básicos en salud (ASIS), en donde la EPOC es la tercera causa de morbilidad en hospitalización con un 2.97%; en la población adulto mayor se evidencia un aumento progresivo de este grupo poblacional (28.1% sexo masculino y 30.3% sexo femenino), esta se encuentra concentrada en edades de 45 a 54 años lo cual es un porcentaje significativo teniendo en cuenta que es una población donde se deben reforzar las acciones de prevención y control de las enfermedades crónicas no trasmisibles; las provincias que más reportan población en este grupo de edad son las del Centro, Sugamuxi y Occidente^8^.

El manejo de la EPOC, se debe realizar de forma integral incluyendo la adopción de medidas farmacológicas y no farmacológicas, con el fin de aumentar la adherencia al tratamiento, disminuir los síntomas y exacerbaciones y, por consiguiente, mejorar la calidad de vida de los pacientes que padecen esta enfermedad^2^. Dentro del tratamiento farmacológico, los broncodilatadores se consideran como la piedra angular del tratamiento sintomático, debido a que producen la relajación del músculo liso bronquial disminuyendo la obstrucción del flujo aéreo; estos de acuerdo a la duración de acción se pueden clasificar en broncodilatadores de acción corta y de acción prolongada y según el mecanismo de acción, en antimuscarínicos y B2-agonistas^9^.

Estos medicamentos son administrados diariamente a través de dispositivos denominados inhaladores^10^; algunos pacientes pueden presentar mala adherencia a la medicación, esta adherencia depende de varios factores entre ellos la calidad y facilidad de uso del dispositivo, lo cual se ve reflejado en el nivel de satisfacción del paciente con el dispositivo empleado^11^.

Existen pocas publicaciones respecto a la satisfacción de los pacientes frente a los dispositivos de inhaloterapia; adicionalmente los pocos instrumentos estandarizados y diseñados para evaluar este aspecto se han elaborado en contextos latinoamericanos diferentes a Colombia^12^; por lo tanto, se vio la necesidad de validar el cuestionario FSI-10 sometiéndolo a pruebas estadísticas para determinar su validez y confiablidad, en pacientes diagnosticados con EPOC de una provincia del departamento de Boyacá-Colombia, y a su vez aplicar el instrumento para determinar la satisfacción del paciente con los dispositivos de inhaloterapia.

## Materiales y Métodos

Se llevó a cabo un estudio transversal prospectivo y validación de contenido. El universo de estudio estuvo conformado por los pacientes diagnosticados con el CIE-10 para EPOC en el periodo 2012-2013 de las Empresas Sociales del Estado (E.S.E.) de primer y tercer nivel de la provincia centro del departamento de Boyacá - Colombia.

Para la validación del instrumento FSI-10 se determinó una muestra de 100 pacientes con diagnóstico de EPOC según Osterlind quien indica que basta con que la muestra este conformada entre 50 y 100 participantes^13^, los cuales contaron con las mismas características sociodemográficas, de diagnóstico y de procedencia que los participantes de la segunda fase del estudo correspondiente a la evaluación de la satisfacción. De otra parte, para determinar la satisfacción de los pacientes con los dispositivos de inhaloterapia a través del cuestionario FSI- 10, posterior a la validación, se estableció un muestreo probabilístico estratificado, la muestra fue diseñada en el programa Epidat teniendo en cuenta una proporción esperada del 8.6% y un nivel de confianza del 95% para una población total de 13.923, teniendo como referente la información que proporciona el Sistema de Información de la Secretaria de Salud de Boyacá SISPRO, sobre la población con diagnóstico de EPOC, obteniendo un tamaño de muestra de 337 sujetos que participaron en el proyecto; para determinar la muestra para cada uno de los municipios se desarrolló el método de afijación proporcional.

Para asegurar la participación equitativa de cada uno de los municipios, se estableció el método de afijación proporcional para cada uno de estos, y a la vez, los sujetos fueron seleccionados de forma aleatoria tomando como base el listado de pacientes con EPOC proporcionado por cada municipio, dicha selección se realizó en el programa Epidat; posteriormente vía telefónica se contactaron los pacientes con el fin de citarlos en una fecha determinada para la firma del consentimiento informado así como para la realización de la prueba de espirometría forzada y la aplicación de los instrumentos respectivos. Estableciendo como criterios de inclusión a los pacientes mayores de 45 años con diagnóstico de EPOC estable, conscientes, alertas y colaboradores para la realización de la prueba espirométrica; como criterios de exclusión se consideraron, los pacientes con exacerbación de la EPOC, con contraindicaciones para la toma de espirometría, con trastorno psiquiátrico y aquellos que participaron en el proceso de validación.

Una vez seleccionados los pacientes participantes se les aplicó el cuestionario FSI-10, el cual es un instrumento que consta de 10 preguntas, cada una con 5 opciones de respuesta en escala Likert con 5 opciones de respuesta: mucho, bastante, algo, poco y muy poco puntuadas respectivamente de 5 a 1 para una puntuación total de 50; este cuestionario evalúa el grado de satisfacción de los pacientes con el aparato de inhalación. El estudio contempló una descripción de variables sociodemográficas, en lo referente a las variables cuantitativas fueron expresadas por medias y desviación estándar.

Inicialmente se llevó a cabo una descripción de las variables sociodemográficas por sexo. La descripción de las variables cualitativas se realizó por medio de frecuencias absolutas, frecuencias porcentuales, para el caso de las variables cuantitativas con una dispersión simétrica estas fueron expresadas por medias y desviación estándar. Se calculó el estadístico Ji^2^ para evaluar la dependencia de las variables sociodemográficas y el sexo. Posterior a esto se realizó evaluación de las *propiedades psicométricas*. El análisis factorial exploratorio (AFE) se realizó por medio del método de componentes principales, con rotación *varimax,* según el grado de correlación existente entre ellos. Se determinó, a través de la prueba de esfericidad de Bartlett, el determinante de la matriz de correlaciones y la prueba de Kaiser-Meyer-Olkin (válido con valores por encima de 0.5) que era aplicable un AFE. De esta manera, la inclusión de cada ítem en un determinado factor, se realizó si existía un grado de saturación mínimo de 0.4 y un *autovalor* mayor de 1^13^. La cantidad de factores se estipuló sin restricción de estructura, y posteriormente, mediante la determinación de un número reducido de factores, según los resultados del *screen test*. Para calcular la confiabilidad se usó el Alpha de Cronbach posterior a la consolidación de los factores, el valor de alfa fuera superior a 0.70^14^, en la comparación entre grupos de sujetos; para la comparación entre individuos se tomó un valor de coeficiente α de Cronbach mayor de 0.90, *ese se estimó* con la puntuación total del cuestionario, también se calcularon las modificaciones de ese valor al excluir cada uno de los ítems del cuestionario. Se tomó una significatividad nominal de 0.05 para todos los cálculos; todos los datos se analizaron con el paquete comercial estadístico y SPSS 23 (Chicago, IL, USA). El proyectó contó con el aval del Comité de Bioética de la Universidad de Boyacá (CB 131). Todos los pacientes firmaron y aceptaron el respectivo consentimiento informado.

Como limitaciones del estudio se refiere que la mayoría de los pacientes administran sus medicamentos a través de dispositivos de dosis medida, muy pocos reportaron el uso de dispositivos como nebulizadores e inhaladores de polvo seco, por lo que no fue posible determinar el dispositivo que genera mayor satisfacción.

## Resultados

Se evaluaron 337 pacientes con diagnóstico clínico de EPOC de los 15 municipios de la provincia centro del Departamento de Boyacá - Colombia que cumplieron los criterios de inclusión, los cuales fueron seleccionados aleatoriamente con una muestra representativa de cada uno de los municipios contando con un 59% de mujeres [IC95% 53.7 - 64.7] y 41% de hombres [IC95% 35.3 - 46.3].

La medicación para el tratamiento del EPOC puede ser administrada a través de diferentes dispositivos como: nebulizador, inhalador de dosis medida de cartucho presurizado e inhalador de polvo seco. Este aspecto fue indagado en la población estudiada y se evidenció que el 100% de los pacientes que reciben tratamiento farmacológico lo hacen por medio del dispositivo inhalador de cartucho presurizado. Así mismo, se evaluó el grado de satisfacción de estos pacientes frente al dispositivo que utilizan a través del cuestionario FSI-10.

**Figura 1:**
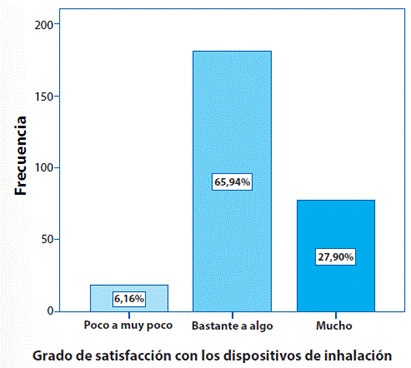
Satisfacción del paciente con EPOC con los dispositivos de inhalación.

La Figura 1, evidencia de acuerdo con la puntuación obtenida en el cuestionario FSI-10, que el 66% (IC95% 60.2 - 71.4) de los pacientes que reciben tratamiento farmacológico con dispositivos de inhalación, en este caso inhalador de dosis medida de cartucho presurizado, se encuentran en un rango entre bastante a algo satisfechos y solo el 28% (IC95% 22.5 - 33.3) están muy satisfechos con el dispositivo.

La Tabla 1**,** evidencia que el coeficiente del alfa de Conbrach obtenido en cada una de las variables y en el instrumento general es excelente (> 0.9), con una fuerza de correlación importante, lo que indica que el cuestionario FSI10 es confiable para determinar la satisfacción de los pacientes frente a los dispositivos de inhalación.

La Tabla 2**,** refiere que el determinante de la matriz fue de 1.54, lo cual indica que se trata de una matriz de identidad; sin embargo, el KMO obtenido es muy cercano a 1 y la prueba de esfericidad de Bartlett reportó 0.000, lo que indica que existe asociación y correlación entre las preguntas del cuestionario FSI10 y así mismo indica que el análisis factorial es aceptable y adecuado, por lo anterior se decidió continuar con el análisis factorial, a pesar del valor obtenido en la determinante de la matriz. Respecto a la varianza total explicada, se determinó como autovalor un valor mayor a 1 para determinar el número de componentes requeridos, lo cual reportó que solo un componente es suficiente y éste aporta un 81% para evaluar la satisfacción de los pacientes que utilizan dispositivos de inhalación.

**Figura 2:**
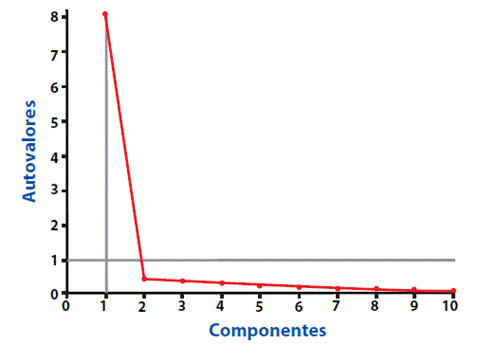
Sedimentación

Lo anterior, se corrobora con el gráfico de sedimentación (Figura 2) en el cual se evidencia que, a partir del segundo componente, de acuerdo a los autovalores, éstos no inciden en mayor medida confirmando que la matriz es una matriz de identidad.

## Discusión

El asma y la EPOC son enfermedades pulmonares obstructivas crónicas que afectan a millones de pacientes y agregan carga a los sistemas de atención médica en todo el mundo. Según las pautas de tratamiento actuales, las dos enfermedades se tratan con medicamentos de mantenimiento administrados diariamente por dispositivos inhalados (generalmente una combinación de broncodilatadores y corticoesteroides). Sin embargo, es común que los pacientes bajo tratamiento prolongado tengan una mala adherencia a la medicación. El manejo farmacológico del paciente con EPOC tiene como objetivo reducir los síntomas, la frecuencia respiratoria, la gravedad de las exacerbaciones, tolerancia al ejercicio y el estado de salud^15^. Los dispositivos para este tratamiento son inhaladores de dosis medida; la adherencia al tratamiento de los pacientes puede verse influida por su estado general, así como por la frecuencia de dosificación, sus expectativas con respecto al tratamiento de su enfermedad y sus resultados; en gran medida por la calidad y facilidad del uso del dispositivo^16^.

De acuerdo a la Global Initiative for Chronic Obstructive Lung Disease (GOLD) se estandarizan los medicamentos administrados por medio de inhaladores; la eficacia de estos determina el éxito de la terapia^17^; entre el 40% y el 60% de los pacientes con EPOC tienen una mala adherencia al tratamiento, relacionada con factores dependientes del paciente, del tratamiento farmacológico y/o factores sociales, por tanto, es importante considerar las características individuales y, de acuerdo con los parámetros, elegir el medicamento y el inhalador de forma personalizada^18^, de acuerdo con este abordaje al paciente de forma integral, instaurándole manejos farmacológicos, según las características propias del individuo y con el fin de aumentar la adherencia al tratamiento y disminuir los síntomas, exacerbaciones y, por consiguiente, mejorar la calidad de vida^19^^,^^9^.

Adicional a lo anterior, se dispone de diversas herramientas psicométricas e instrumentos cronométricos que permiten evaluar indicadores no biológicos o directamente observables como la satisfacción con los dispositivos de inhalación empleados en el tratamiento farmacológico de las patologías pulmonares^20^. A pesar de ello, y la escasa cantidad de artículos científicos que han utilizado dichos instrumentos en el ámbito mundial, lo referente al proceso de construcción y validación de escalas de medición en salud sigue presentando limitaciones relacionadas con la falta de claridad en algunas comunidades académicas sobre los criterios que deben evaluarse, la ausencia de consenso sobre el método de construcción y validación de las escalas, y la diversidad de opciones metodológicas con que se llevan a cabo estos procesos^21^, ^22^, ^23^, ^24^. En este orden de ideas, se considera que la ausencia de equivalencia entre las diferentes escalas, derivada de un proceso de validación deficiente, reduce las posibilidades de hacer comparaciones entre poblaciones de diferentes países, culturas e idiomas, impidiendo el intercambio de información en la comunidad científica.

En el área de ciencias de la salud, la medición del estado de los individuos se ha hecho desde la perspectiva biomédica del proceso salud-enfermedad, mediante el uso de marcadores biológicos denominados desenlaces duros u objetivos; es por esto que en las áreas de la salud es necesario disponer cada vez es más de instrumentos de medida, que permitan evaluar atributos subjetivos que integran constructos y dimensiones más complejas, como medio para orientar acciones de atención, promoción o protección de la salud^25^. El empleo de formularios estandarizados ofrece una alternativa fiable, siempre que su proceso de elaboración o la tarea de traslación transcultural hayan sido metodológicamente correctos^26^.

Hasta la fecha, se ha documentado escasamente a cerca de la preferencia de pacientes para el uso y administración de los diferentes dispositivos inhalados; además, los pocos instrumentos estandarizados diseñados específicamente para analizar este elemento en particular, se ha desarrollado en el ámbito cultural; contextos que difieren de los desarrollados en España^27^^,^^28^ quizás abordando y explicando la muy limitada contribución a la literatura en esta área^29^^,^^30^; en un esfuerzo por abordar estas limitaciones, uno de los autores del presente estudio^31^ desarrolló un cuestionario español, denominado FSI-10 (Sensación de satisfacción con el inhalador) instrumento auto administrado, el cual está diseñado para determinar la facilidad de uso y la satisfacción que refiere el paciente en relación con los dispositivos inhalados, independientemente del medicamento utilizado. Según los autores del cuestionario, tiene una consistencia interna aceptable y un buen test-retest de fiabilidad, pero no se especificó la diferencia significativa o sensibilidad al cambio, indicando que el cuestionario es comprensible y de fácil aplicación, dado que posee propiedades psicométricas adecuadas; dada su unidimensionalidad, el cuestionario ofrece una puntuación que resume los diversos aspectos que los pacientes expresan en el nivel de satisfacción (simplicidad de aprendizaje, facilidad de uso, portabilidad, entre otras características)^25^.

Respecto a la satisfacción con el tratamiento, definido como la evaluación del proceso del tratamiento y sus resultados asociados, en este estudio se evidenció que los pacientes reciben tratamiento farmacológico con inhalador de dosis medida de cartucho presurizado, encontrándose en un rango entre bastante a algo satisfechos, solo el 28% (IC95% 22.5 - 33.3) están muy satisfechos con el dispositivo; Eleftherios Zervas y Col. en su estudio con una cohorte de pacientes griegos, identificaron que los pacientes con EPOC grave expresaron una mayor satisfacción que en aquellos con enfermedad leve o moderada, independientemente del dispositivo utilizado.

En este estudio se realizó la validación del cuestionario FSI 10 diseñado por Perpiña Todera *at Col*^25^ para evaluar la satisfacción del paciente con el uso de sistemas de inhalación en la población con diagnóstico de EPOC de la provincia Centro del Departamento de Boyacá.; Perpiña et al en su estudio realizaron pruebas psicométricas a este instrumento, reportando que el alfa de Cronbach de la totalidad del cuestionario fue satisfactoria con un resultado de (0.92), dato similar al obtenido en el presente estudio, cuyo valor fue de (0.96).

Eleftherios Zervas *et al*, en su estudio^16^ aplicaron la versión griega del cuestionario FSI 10 para evaluar la satisfacción de los pacientes con el dispositivo de inhalación que usaban como parte de su tratamiento; los autores refieren que el cuestionario fue fácilmente comprendido y completado por los participantes. Ninguno de los diez ítems del cuestionario quedó sin respuesta. El FSI-10 mostró una homogeneidad adecuada sin redundancia, ya que no se encontró correlación >0.8 entre las preguntas. La prueba de Cronbach para el cuestionario en su conjunto mostró muy buena consistencia interna (α de Cronbach = 0.898), lo cual evidencia semejanza con los datos obtenidos al analizar las propiedades psicométricas del instrumento en el presente estudio.

Por otra parte, el cuestionario FSI-10 también se ha utilizado para evaluar la satisfacción de 442 pacientes con EPOC en Turquía, en la práctica clínica de la vida real; los datos sobre la facilidad de uso y portabilidad del dispositivo se recopilaron sobre la base de una única visita de paciente, refiriendo que la mayoría de los pacientes utilizaron dispositivos multidosis de polvo seco, mientras que la edad avanzada se asoció con una mayor tasa de errores en las maniobras de inhalación, mientras que la mayoría de los pacientes estaban satisfechos con el uso general del dispositivo^32^, datos que son comparables con nuestro estudio el cual fue aplicado igualmente en pacientes de atención primaria, los cuales reportaron bastante o algo de satisfacción con los dispositivos de inhaloterapia; de igual forma, el estudio realizado por Niño *et al*^33^, desarrollado en uno de los municipios de la provincia centro, reporta que el grado de satisfacción es entre bastante a algo, lo cual es equivalente a los resultados del presente estudio.

Nuestras estimaciones sobre la validación del cuestionario FSI-10 indican que es un instrumento comprensible y fácil de manjar, el cual presenta propiedades psicométricas bastantes satisfactorias; dada su unidimensionalidad, ofrece puntuaciones resumen de los diferentes aspectos que evalúan la satisfacción del paciente con un dispositivo concreto con base en la disponibilidad de uso, comodidad de manejo, facilidad de transporte, situación que infiere en lo descrito por Guyatt *et al*^34^ en su estudio que evaluó la satisfacción y preferencia de los pacientes por dispositivos de inhalación.

## Conclusión

Se considera que el cuestionario FSI-10 es un instrumento válido para evaluar el grado de satisfacción del paciente con EPOC que utiliza dispositivos inhalados, teniendo en cuenta que es comprensible, de fácil manejo y que exige comprensibilidad en los ítems evaluados.

La satisfacción de los pacientes con EPOC en el uso de los dispositivos para la administración de la medicación inhalada no es plena en la totalidad de los participantes, por tanto, es necesario considerar reevaluar el dispositivo por aquel que genere mayor satisfacción, con el fin de alcanzar la adherencia al tratamiento.

**Tabla 1:** Propiedades psicométricas del cuestionario FSI10

	Varianza de la escala si se elimina el elemento	Correlación elemento -total corregida	Alfa de Cronbach si se elimina el elemento
Factor I			
MOS 14	46,720	0,708	0,903
MOS 18	47,920	0,686	0,905
MOS 7	48,033	0,674	0,905
MOS 11	46,871	0,721	0,902
MOS 10	45,917	0,708	0,903
MOS 19	45,971	0,733	0,901
MOS 8	47,679	0,702	0,904
MOS 16	44,937	0,721	0,902
MOS 20	45,683	0,660	0,907
Sub-total			0,913

Fuente: Las autoras

**Tabla 2:** Varianza total explicada

	Autovalores iniciales			Sumas de las saturaciones al cuadrado de la extracción		
	Total	% de la varianza	% acumulado	Total	% de la varianza	% acumulado
Factor 1	8,073	80,730	80,730	8,073	80,730	80,730
Análisis						
Factorial						
Índice KMO	0.953					
Prueba de Barlett (Ji2)	3644.8					
Grados de libertad	45					
Significancia	0.000					

KMO: Índice de adecuación de Kaiser - Meyer - Olkin. Método de extracción: Análisis de Componentes principales

Fuente: Las autoras

Fuente: Las autoras

Fuente: Las autoras

### Aspectos éticos

El proyecto fue presentado al Comité de Bioética de la Universidad de Boyacá el cual dio su aval para el desarrollo del mismo; en la documentación presentada se explicó que la investigación sería de riesgo mínimo de acuerdo establecido en la Resolución 008430 del 04 de octubre de 1993 del Ministerio de Salud de la República de Colombia. Así mismo previo a la toma de datos se explicó a cada uno de los pacientes la finalidad de la investigación y su participación en ella informando detalladamente los riesgos y beneficios a los cuales estarían expuestos y los datos que debían reportar, así como también el cuestionario a aplicar. Posterior a ello, los pacientes que aceptaron participar del estudio firmaron el respectivo consentimiento informado.
